# Development and application of a relative soil water content – transpiration efficiency curve for screening high water use efficiency wheat cultivars

**DOI:** 10.3389/fpls.2022.967210

**Published:** 2022-08-26

**Authors:** Yunzhou Qiao, Dongxiao Li, Wenjun Qiao, Yongpeng Li, Hong Yang, Wenwen Liu, Mengyu Liu, Xiying Zhang, Baodi Dong

**Affiliations:** ^1^Key Laboratory of Agricultural Water Resources, Hebei Key Laboratory of Agricultural Water-Saving, Center for Agricultural Resources Research, Institute of Genetics and Developmental Biology, Chinese Academy of Sciences, Shijiazhuang, China; ^2^State Key Laboratory of North China Crop Improvement and Regulation/Key Laboratory of Crop Growth Regulation of Hebei Province, College of Agronomy, Hebei Agricultural University, Baoding, China

**Keywords:** *TE–RSWC* curve, power function, transpiration efficiency, yield, water use efficiency

## Abstract

Improving water use efficiency (*WUE*) has been proven to be a prosperous way to produce more grain in drought-prone areas. Transpiration efficiency (*TE*) has been proposed as a criterion for screening cultivars with high *WUE*. This study quantifies the relations of TE to relative soil water content (*RSWC*) gradients using pot experiments and evaluates the capability of the relations of *TE*-*RSWC* on assessing the cultivar performance in field yield and *WUE*. Twelve winter wheat cultivars were grown at 6 *RSWC*, 12.1, 24.2, 36.3, 48.4, 60.5, and 72.6% of field capacity (*FC* = 24.8 g/g) for 33 days in tightly sealed pots preventing soil evaporation. The results showed that *TE* decreased power functionally following the increase in *RSWC* for all cultivars. The relationship could be described as *TE* = *TE _*FC*_* × (*RSWC*) *^b^*, named *TE*–*RSWC* curve. This curve could be divided into an orderly area where the rank of cultivars was stable when *RSWC* ≤ 12.1% or *RSWC* ≥ 72.6% and a disorderly area where the rank was unstable when 12.1% < *RSWC* < 72.6%. To assess the consistency of pot TE to field yield and *WUE*, the same 12 varieties were grown under rainfed and two irrigations (75 mm at the jointing and flowering stages, respectively). *TE _*FC*_* was found to be positively related to field yield and *WUE* independent of irrigation. TE measured near the wilting point was negatively related to field yield and *WUE*. These results indicated that *TE _*FC*_* could be used as a surrogate for screening high-yield and high-*WUE* cultivars. The consistency and inconsistency can be attributed to the orderly area and disorderly area of the *TE*–*RSWC* curves.

## Introduction

Wheat (*Triticum aestivum* L.) is the major food crop in most countries worldwide. Most wheat crops are planted in arid and semiarid regions or during drought seasons. The scarcity of freshwater resulting from global climate change, population growth and urbanization is making irrigation more difficult than ever (Shiferaw et al., 2013). Thus, wheat production is usually restricted and will continue to be limited more seriously by drought stress (Lobell et al., 2015). However, wheat demand is expected to increase by over 30% due to human population growth (Prasad et al., 2011). Therefore, how to produce more grain per drop has already been intensively studied in drought-prone areas. Improving wheat water use efficiency (*WUE*, termed grain or biomass production per unit of water use) is considered a possible method (Tfwala et al., 2021). Breeding for varieties with high transpiration efficiency (*TE*, biomass production per unit of transpiration) may help to achieve high *WUE* cultivars. However, the ability to screen cultivars with high *TE* from large populations is limited by slow, low-throughput, and/or expensive screening procedures (Fletcher et al., 2018).

Many properties are known to be suitable as surrogates for high *WUE* and yield. Stable isotope ^13^C is discriminated during fixation of carbon and thus plants contain a smaller ratio of ^13^C to ^12^C than the atmospheric CO_2_ [Carbon isotope discrimination (Δ)]. Δ has been observed tightly related to *WUE* and *TE* and thus widely accepted as an indicator for *WUE* (Farquhar and Richards, 1984; Condon et al., 1993; Deery et al., 2016). It has been used to select cultivars with high *WUE* and as a marker in breeding assisting in phenotypic selection for high *WUE* (Collard et al., 2005). Canopy temperature reveals the ability of plant to extract soil water and varies among genotypes due to variations in energy balance, stomatal conductance and transpiration (Balota et al., 2008). Lu et al. (2020) evaluated the possibility of using canopy temperature as an indicator to improve the yield and *WUE* of winter wheat and concluded that the ability to efficiently use soil water and maintain dry matter production at the grain-filling stage are the two important desired characteristics. Other possible traits, such as n-alkanes in leaf wax (the first or second abundant component in leaf wax, Wang et al., 2015; Liu et al., 2019), ash content (significantly correlated to 1/*TE* and Δ, Masle et al., 1992), chlorophyll content, photosynthetic rate, and *TE* at the leaf and plant levels (These characters are directly related to carbon fixation and WUE, Misra et al., 2010; Deery et al., 2016), are also related to grain yield and *WUE*. Among all traits studied, *TE* at the plant level (g mass/kg transpiration) is considered suitable as an indicator of yield and *WUE* due to its stability and accessibility in comparison with *TE* at the leaf level.

At the plant level, *TE* is the ratio of biomass yield (*BY*) per unit of transpiration loss (*Tr*). *WUE* is the ratio of *BY* to evapotranspiration (*ET*). Here, *ET* is composed of non-productive evaporation from the soil surface (*E*) and productive *Tr* through the crop surface. *E* is an important component of *WUE* but is excluded when referring to *TE*. Therefore, *TE* is more like an intrinsic feature of a certain cultivar than *WUE*. A three-factor model was introduced to indicate the importance of *TE* in relation to *WUE* (Ehdaie, 1995): *WUE* = *UE* × *TE* × *HI*, where *UE* is the ratio of water transpired to seasonal *ET*, *HI* is the harvest index, and *TE* is the ratio of biomass to transpiration. Although the function indicated that choosing cultivars with high *TE* may play an important role in improving the *WUE*, the question remains of how to measure and compare *TE* between varieties.

The difficulty in accurate measurement of wheat *TE* exists in that *E* and *Tr* cannot be accurately separated from *ET* (Tfwala et al., 2021). *ET* is usually determined by the soil water balance equation (Function 4 in the section “Materials and methods”) using soil cores, neutron probes or soil water probes. *Tr* is not easy to measure directly and is usually calculated as *ET*-*E*, and *E* is measured using a lysimeter. In the situation of a micro lysimeter, *E* is usually overestimated with the absence of root water uptake, which causes a higher soil water content (*SWC*) in the lysimeter than that around the root system (Villalobos and Fereres, 1990). In the situation of a large lysimeter, the soil surface is usually covered by particles and beads to prevent the occurrence of *E*, and the total water loss is considered *Tr*. For example, Vadez et al. (2008) used large pots (0.2 m diameter and 1.0 m high for groundnut and chickpea; 0.25 m diameter and 2.0 m long for pigeon pea) to make the situation similar to field conditions. However, only 90% of soil evaporation is prevented with a 2 cm polyethylene bead layer. Fletcher et al. (2018) provided a small pot-in-bucket method to rapidly and accurately screen for high *TE* wheat cultivars. However, the shortcoming of the pot-in-bucket method is that only one soil moisture can be formed due to the method of water supplementation.

The rank of *TE* among cultivars usually varied under different soil moistures. This aroused the question of how to compare *TE* between genotypes. Studying the relationship between *TE* and *SWC* may help us to understand the variation in the rank of cultivars. To date, scientists have checked the effect of soil moisture (Siahpoosh and Dehghanian, 2012; Natarajan et al., 2021) on the *WUE* and/or *TE* of various kinds of crops. In addition to the negative linear regression of *TE* against *ET* reported by Siahpoosh and Dehghanian (2012), the response of *TE* to gradients of *SWC* has been less studied. The present study offered a method to study the relations of *TE* to *SWC*. The objectives of the present study were (I) to assess the response pattern of *TE* along *SWC* gradients and (II) to evaluate whether *TE* measured at the seedling stage can help to screen wheat cultivars with high yield and *WUE* in the field.

## Materials and methods

### Study area

The present study included two experiments, a pot experiment and a field experiment. They were conducted at the Luancheng Agro-Ecosystem Experimental Station of the Chinese Academy of Sciences (114° 40′ E, 37° 50′ N, 50.1 m a.s.l). The experimental station is located on the North China Plain (NCP), which is characterized by a winter wheat–summer maize annual double-cropping system, and water shortages occur during the winter wheat-growing season. The annual precipitation varies be-tween 400 and 600 mm, and approximately 30% falls in the winter wheat-growing season on the NCP.

### Pot experiment

**FIGURE 1:**
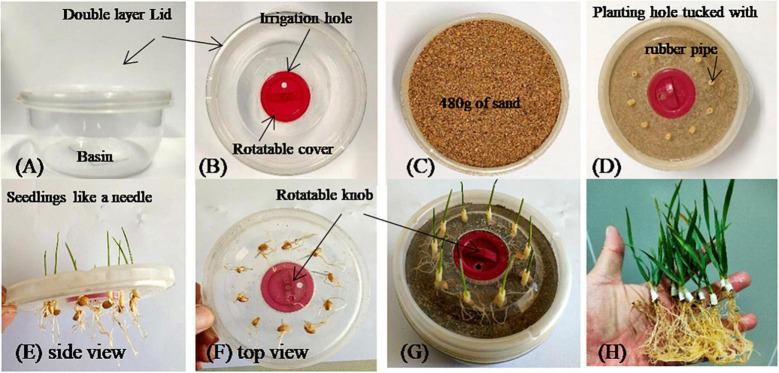
The technological process of the pot experiment evaluating wheat *TE* response to gradients of soil water contents. The top view and side view of the pot is shown in panels **(A,B)**. The pot is filled with sand **(C)**. The lid was placed on the pot with sand after planting holes were tucked with rubber pipe 6 ∼ 8 mm long **(D)**. Seedlings were tucked into the rubber pipe after growing into a needle shape in a petri dish **(E,F)**. The pot was covered with lid with seedlings after being watered 1 higher above the target soil moisture **(G)**. The pot was broken using hacksaw for taking photograph **(H)**. If a whole plant is not needed, the seedlings could be cut off outside the pot using scissors first, and continue to be cut into aboveground and belowground parts from the node between root and shoot, after removing the lid.


A pot experiment was conducted to screen wheat cultivars with high *WUE* from their *TE* at the seedling stage. Twelve cultivars were exposed to 6 *RSWC* levels. Each combination was duplicated 4 times. The experiment was conducted in the following process:

(1) About the pot. The plastic pot used in the present study was a product of an invention patent of the authors, Figure 1 (Liu and Liu, 2006). The pot (*d* = 110 mm, *h* = 50 mm) was composed of a basin part and a double layer lid. The two parts made a whole pot when the lid was tightly clung to the basin. The double layer lid included a bottom layer and an upper layer. The bottom layer (*d* = 110 mm) was the same diameter as the basin. The upper layer was a rotatable cover (*d* = 35 mm). The two layers were fixed together through an axis at the center of the bottom layer. There is a rotatable knob at the center of the upper lid layer. The upper layer could be turned by turning the knob. Irrigation holes (*d* = 3 mm), 15 mm away from the axis, were made on both layers of lid. Researchers could provide water through the irrigation hole or keep the pot sealed by rotating the upper layer.
(2) Preparation of seedlings. Approximately 500 seeds of each cultivar were pre-soaked on November 1, 2020, in pure water for 24 h and then placed on a piece of filter paper in a petri dish. The seeds were covered with another piece of filter paper to maintain humidity around the seeds. The seedlings were suitable for the experiment when the buds grew up to 20 ∼ 30 mm long.
(3) Preparation of pots. Ten seedling holes (seedling hole, *d* = 4 mm, 20 mm from the edge and 35 mm to the center) were made equidistantly from each other using a portable electric drill. The holes were then filled with a rubber tube (6 ∼ 8 mm long, 4 and 3 mm of outer and inner diameter, respectively). The pot was then filled with tap-water-washed and air-dried sand that passed through 60 mesh sieves. The total weight of each pot was recorded as W0. The field capacity (*FC*), wilting point (*WP*) and maximal available water (*AW*) of the sand were 24.7, 1.2, and 23.5%, respectively.
(4) Transplanting. Seedlings 2 ∼ 3 cm long were tucked into rubber tubes in seed-ling holes on November 5, 2020. Simultaneously, 19.2, 33.6, 48.0, 62.4, 76.8, and 91.2 g of tap water were given to each pot (the corresponding sand moisture was 4, 7, 10, 13, 16, and 19%, respectively) (water weight/dry sand weight × 100%). The double layer lid with seedlings was then tightly fixed together with the basin part, and the irrigation hole was covered.
(5) Growing conditions. Plants were cultivated in a growth chamber system (SAFE-LED-3, Zhejiang, China). The available ground surface was approximately 10 m^2^ (3.6 m long and 2.8 m wide), and the height was 3.5 m in each chamber. Pots with the same cultivar were placed together in a rectangular shape. In each cultivar group, four pots of the same *SWC* were grouped in line, and then all pots were arranged in a sequence of increasing SWC (4 pots of the same *SWC* × 6 lines with increasing *SWC*). Air temperature (18°C/8°C, day/night, 14/10 h), relative air humidity (60%) and light intensity (PAR, 500 μmol photon⋅m^–2^⋅s^–1^; total light intensity, 50000 lux) were controlled automatically. The air entered the chamber from the holes on the four walls of the chamber (*d* = 2 mm, 0.25 mesh). The cold light source was a shadowless design. Therefore, the growing conditions were uniform everywhere in the chamber. There was no need to consider the present experiment a split-plot design. No nutrients were given during the experimental duration.
(6) Water control: Six soil moistures, 12.1, 24.2, 36.3, 48.4, 60.5, and 72.6% of *FC* (equal to absolute *SWC* 3% ± 1%, 6% ± 1%, 9% ± 1%, 12% ± 1%, 15% ± 1%, 18% ± 1%, respectively), were applied in the present study. The sand moisture was measured by weighing the pots once a day using a balance (BSA2202S, Sartorius, Germany). Seedling fresh weight was ignored when deciding to give water or not. The sand moisture was controlled target absolute *SWC* ± 1.0% around the target by giving water to each pot when the moisture was approaching “target absolute *SWC* - 1.0%” until it reached “target absolute *SWC* + 1.0%,” using a 20 ml syringe. No water was given to the 12.1% treatment since *SWC* was approaching the lower limit at the end of the experiment. Two irrigations were applied to the 24.2 and 36.3% treatments, three to the 48.4% treatment and four to the 60.5 and 72.6% treatments.
(7) Harvesting. The pot experiment was harvested 33 days after being transplanted to pots (December 8, 2020, approximately 5 leaves). The pot weight was recorded as W1 using a balance (BSA2202S, Sartorius, Germany). The seedlings were cut off from the pot surface, and then the lid was removed. The remaining shoot was then separated from the root at the tillering node. The root was washed free of sand. The shoots and roots were then oven-dried at 80°C.
(8) Pot *TE* calculation. Total final weight, including dry shoots (W2) and dry roots (W3), was measured. *TE* was calculated as

(1) TE=total(shootmass+rootmass)/Tr

where shoot mass is dry shoot weight; root mass is root dry weight; and Tr is transpiration, which is calculated as the total irrigation amount minus the water left in the pot (W1 − W0 − W2 − W3 + irrigation). *TE* total was also divided into *TE* shoot and *TE* root. They were calculated as the ratio of dry mass produced by each part to the total water loss.


(2) TE=shootshootmass/Tr

(3) TE=rootrootmass/Tr

### Field experiment

#### Experimental design

To assess the consistency of seedling *TE* to field-measured *WUE* of a whole growing season, the 12 winter wheat cultivars were also grown in a field at the Luancheng Experimental Station. The field experiment was completely a randomized de-sign. Cultivars and irrigation were randomly assigned to each plot. The experiment was repeated 3 times, and 72 plots were set up (1.4 m × 10 m of area, 8 rows with 17.5 cm of row space). The protection line was 1.0 m between plots planted with the wheat Cultivar Shimai 19. Half of the 72 plots were given no water, and another half were given two irrigations (75 mm at the jointing stage and 75 mm at the flowering stage). The field experiment was sowed using a plot planter (ZZXB-8A, Haoqing Machinery Factory, Hebei, China).

**FIGURE 2:**
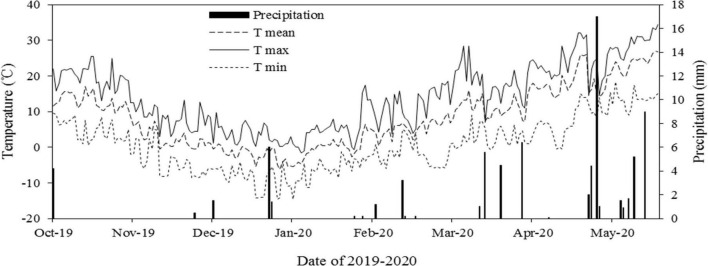
Meteorological conditions during the field experiment from October 16, 2019 to June 10, 2020.

The field had a loamy soil with 1.2% organic matter and with 15 mg kg^–1^, 150 mg kg^–1^, and 80 mg kg^–1^ available phosphorus, potassium, and nitrogen, respectively. Before rotary tillage, 220 kg P_2_O_5_ ha^–1^, 210 kg N ha^–1^, and 45 kg K_2_O ha^–1^ were given as base fertilizers, and there was no top dressing during the whole growing period. Wheat was sown on October 15, 2019. The soil water content at sowing time was 73.6% ± 3.5% of the field capacity in the 0 cm ∼ 20 cm soil layer. The meteorological conditions of the wheat-growing season are shown in Figure 2.

#### Calculation of water use efficiency

An area of 4 rows × 2 m (1.40 m^2^) in nearly the middle of each plot was harvested manually at physiological maturity on the 5th (drought treatment) and 10th (irrigated treatment) of June 2021. Grain was obtained using a threshing machine (QKT-320A, Weihui Xinnongke Machinery Factory, Henan, China) and air-dried to constant weight. The SWC at sowing and harvesting time was measured every 10 cm from ground sur-face down to 200 cm by soil cores and oven (DHG-9620A, Shaying Scientific Instrument Co., Ltd., Shanghai, China) dried to a constant weight at 60°C.

*ET* was calculated according to the water balance equation (Allen et al., 1998):


(4) ET=SWD+P+I-D+CR-R

where, *SWD* is soil water depletion from 0 to 200 cm profile (initial soil water content minus final soil water content), *P* is precipitation, *I* is irrigation, *D* is drainage from the root zone, *CR* is capillary rise to the root zone, and R is runoff. Equation (4) was simplified by zeroing 3 terms: (1) *D* = 0 since irrigation and precipitation were not high enough to leak below 200 cm. (2) *CR* = 0 due to the deep groundwater table (ap-proximately 40 m below the surface). (3) *R* = 0 as a result of the deep soil profile and large water-holding capacity of the field (Zhang et al., 2008, 2009). As a result, the water balance equa-tion transformed to


(5) ET=SWD+P+I

*WUE* was calculated as GY and BY divided by evapotranspiration (ET),


(6) WUE=GY/ET

(7) WUE=BY/ET

### Data analysis

Statistical analysis was performed in SPSS 17.0 (Statistical Product and Service Solutions, IBM). The regression of *TE* to *RSWC* was analyzed as


(8) TE=TE×FCRSWCb

where, *TE _*FC*_* and b are constant items. *TE _*FC*_* is the minimal potential *TE* of a certain cultivar since wheat is seldom planted under conditions of soil moisture higher than *FC*. The *TE*–*RSWC* curve was drawn in Microsoft Office Excel 2013 (Microsoft Co., Ltd.). The Pearson correlation and linear regression of *TE* (at different *RSWC* levels) to field yield and *WUE* of *TE _*FC*_* to b were also analyzed. Two-way ANOVA was applied to test (I) the effect of soil moisture levels, cultivars and their interaction on pot *TE* and (II) the effect of irrigation, cultivars and their interaction on field yield and *WUE*.

## Results

### Growth, transpiration and transpiration efficiency

**FIGURE 3:**
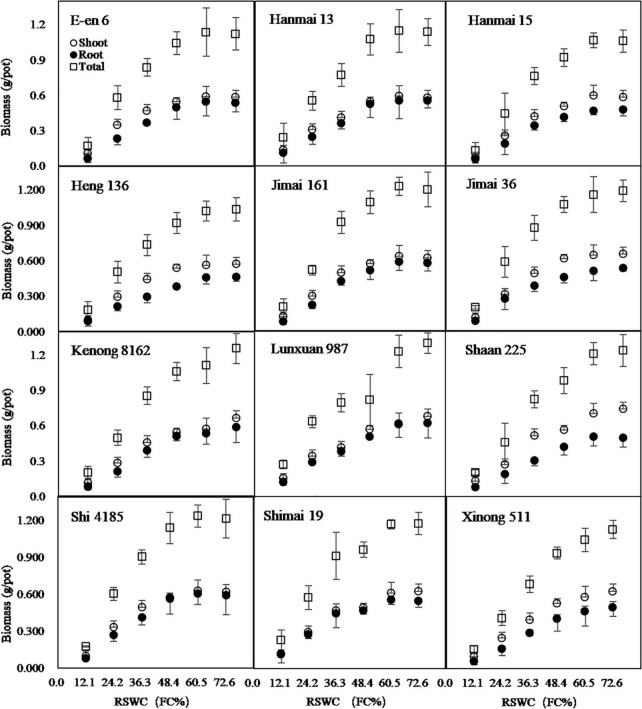
Growth of shoot, root and total biomass after 33 days cultivation under 6 soil water contents. *RSWC*, soil water content. *FC*, field capacity. Data are shown as Mean ± SD, *n* = 4.

Wheat seedlings grew well and showed a significant difference in final biomass at 33 days after exposure to 6 soil moisture levels (Figure 3). Both cultivars and *RSWC* showed significant effects on the growth of wheat seedlings (Table 1). The combination of cultivars and *RSWC* showed a significant interactive effect on shoot dry mass and no effect on root and total dry mass (Table 1). The shoot, root and total biomass per pot (Figure 3) showed an increasing trend with increasing *RSWC*. The increase was in a linear relationship with the increase in *RSWC* from 12.1 to 60.5%. Eight cultivars stopped increasing when the *SWC* reached 60.5%. However, the masses of Heng136, Kenong8162, Lunxuan987 and Xinong511 continued to increase after 60.5% *SWC*.

**TABLE 1:** Main effects of cultivar and *SWC* on seedling growth (*Shoot*, shoot mass; *Root*, root mass; *Total*, total biomass), Transpiration (*Tr*), and transpiration efficiency (*TE*_shoot_, *TE _*root*_*, *TE _*total*_*, the ratio of *Shoot*, *Root* and *Total* to *Tr*).

Treatments		*Shoot* g/pot	*Root* g/pot	*Total* g/pot	*Tr* g/pot	*TE shoot* g/kg	*TE root* g/kg	*TE total* g/kg
Cultivar	Er-en 6	0.44 ± 0.19^cd^	0.37 ± 0.19^b^	0.82 ± 0.38^a^	75.62 ± 45.80^bc^	7.42 ± 3.19^ab^	5.47 ± 1.03^cd^	12.89 ± 4.20^abc^
	Hanmai 13	0.43 ± 0.18^cde^	0.39 ± 0.19^ab^	0.82 ± 0.37^a^	66.54 ± 38.63^cde^	7.70 ± 2.68^a^	6.53 ± 1.49^ab^	14.24 ± 4.17^ab^
	Hanmai 15	0.41 ± 0.21^e^	0.32 ± 0.17^c^	0.73 ± 0.37^b^	65.99 ± 43.88^de^	7.73 ± 2.82^a^	5.98 ± 20abc	13.71 ± 4.80^ab^
	Heng 136	0.42 ± 0.19^de^	0.32 ± 0.15^c^	0.73 ± 0.34^b^	68.92 ± 39.24^cde^	7.06 ± 2.05^ab^	5.34 ± 1.71^cd^	12.40 ± 3.73^bc^
	Jimai 161	0.46 ± 0.20^abc^	0.4 ± 0.21^ab^	0.86 ± 0.41^a^	78.80 ± 47.41^a^	7.06 ± 2.48^ab^	5.66 ± 1.08^bcd^	12.72 ± 3.55^bc^
	Jimai 36	0.47 ± 0.22^ab^	0.38 ± 0.17^b^	0.85 ± 0.39^a^	76.01 ± 43.19^bc^	7.14 ± 1.86^ab^	5.64 ± 1.41^bcd^	12.78 ± 3.25^bc^
	Kenong 8162	0.44 ± 0.20^bcd^	0.39 ± 0.20^ab^	0.83 ± 0.40^a^	68.75 ± 41.62^cde^	7.48 ± 2.04^ab^	6.20 ± 1.06^abc^	13.68 ± 3.09^ab^
	Lunxuan 987	0.46 ± 0.20^bcd^	0.42 ± 0.20^ab^	0.84 ± 0.38^a^	67.27 ± 35.84^e^	7.60 ± 1.52^a^	6.87 ± 0.79^a^	14.49 ± 2.28^a^
	Shann 225	0.49 ± 0.24^a^	0.33 ± 0.17^c^	0.82 ± 0.41^a^	78.33 ± 51.83^a^	7.62 ± 2.72^a^	4.97 ± 1.43^d^	12.59 ± 4.12^bc^
	Shi 4185	0.46 ± 0.21^abc^	0.42 ± 0.21^a^	0.88 ± 0.42^a^	79.22 ± 45.27^a^	6.66 ± 1.76^ab^	5.82 ± 1.07^bcd^	12.48 ± 2.82^bc^
	Shimai 19	0.44 ± 0.20^cde^	0.40 ± 0.17^ab^	0.84 ± 0.37^a^	72.05 ± 38.93^cd^	6.50 ± 0.87^b^	6.20 ± 1.25^abc^	12.71 ± 2.10^bc^
	Xinong 511	0.41 ± 0.21^de^	0.31 ± 0.18^c^	0.73 ± 0.38^b^	70.96 ± 42.77^bcd^	6.83 ± 1.98^ab^	4.86 ± 0.87^d^	11.70 ± 2.84^c^
	F value	5.67**	8.65**	6.69**	8.24**	2.15*	3.57**	2.06*
RSWC (FC%)	12.1%	0.11 ± 0.02^e^	0.09 ± 0.02^e^	0.20 ± 0.04^e^	11.20 ± 2.49^f^	10.92 ± 1.54^d^	7.94 ± 0.86^d^	18.85 ± 1.83^d^
	24.2%	0.30 ± 0.03^d^	0.23 ± 0.04^d^	0.53 ± 0.07^d^	35.67 ± 4.82^e^	8.53 ± 0.72^d^	6.50 ± 0.67^d^	15.02 ± 1.14^d^
	36.3%	0.46 ± 0.04^c^	0.37 ± 0.05^c^	0.82 ± 0.08^c^	65.54 ± 7.30^d^	7.04 ± 0.44^d^	5.67 ± 0.81^cd^	12.70 ± 1.06^d^
	48.4%	0.55 ± 0.03^b^	0.47 ± 0.06^b^	1.00 ± 0.09^b^	93.36 ± 7.37^c^	5.93 ± 0.28^d^	5.16 ± 0.73^c^	11.10 ± 0.92^c^
	60.5%	0.61 ± 0.04^a^	0.53 ± 0.05^a^	1.15 ± 0.07^a^	110.14 ± 9.72^b^	5.64 ± 0.42^b^	4.89 ± 0.67^b^	10.53 ± 1.02^b^
	72.6%	0.63 ± 0.05^a^	0.54 ± 0.05^a^	1.17 ± 0.08^a^	118.32 ± 9.85^a^	5.35 ± 0.42^a^	4.62 ± 0.60^a^	9.98 ± 0.95^a^
	F value	812.81**	360.38**	669.88**	1124.31**	79.59**	31.46**	72.44**
Cultivars × RSWC	F value	1.47*	0.65NS	0.81NS	1.91**	0.81NS	0.34NS	0.47NS

* and ** means main effect is significant at the *P* = 0.05 and *P* = 0.01 levels, respectively. NS means non-significant at the *P* = 0.05 level. Data shown in Cultivar treatment is average over the 6 soil water levels; Data shown in Irrigation treatment is average over the 12 cultivars. Different letters in the same column mean significant differences between treatments at 0.05 level.

**FIGURE 4:**
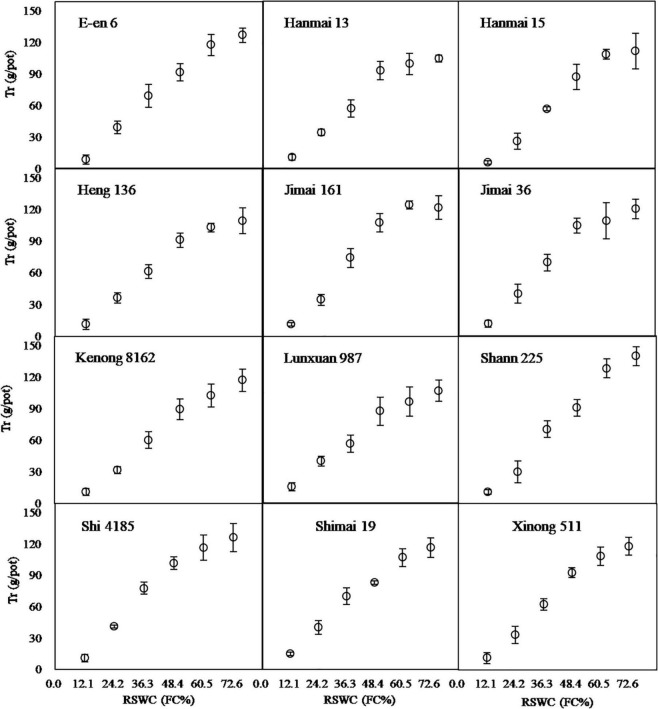
Transpiration per pot after 33 days cultivation under 6 soil water contents. *RSWC*, soil water content. *FC*, field capacity. Data are shown as Mean ± SD, *n* = 4.

Cultivars, *RSWC* and their combination showed a significant effect on Tr (Table 1). The Tr per pot showed a trend of increasing with increasing *RSWC* (Figure 4). The in-creasing trend stopped at a *RSWC* of 48.4% in the Hanmai13 and Jimai36 cultivars and at a SWC of 60.5% in the Hanmai15, Heng136 and Jimai161 cultivars. The other 7 cultivars still showed an uptrend even when the *RSWC* reached 72.6%. These results indicated that these cultivars might not have water saving potential compared to the other 5 cultivars.

**FIGURE 5:**
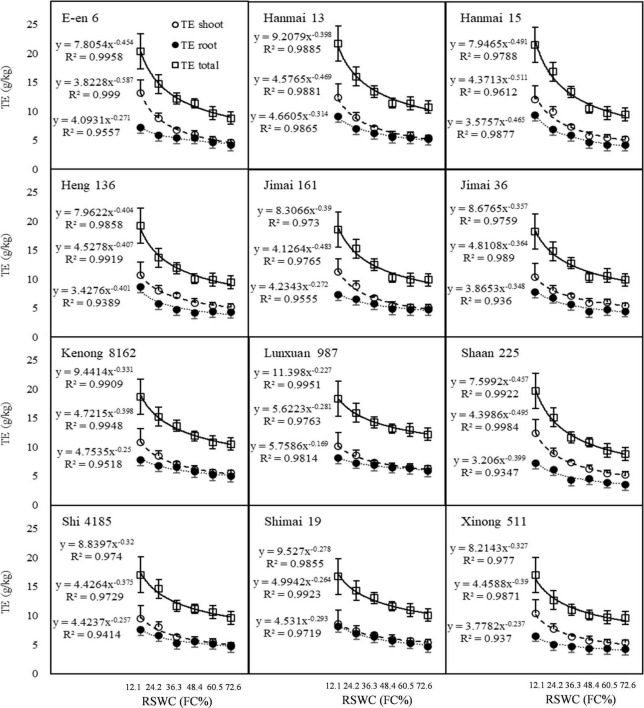
Transpiration efficiency changes following the gradients of soil water content. *TE _*shoot*_*, *TE _*root*_*, *TE _*total*_*, the ratio of shoot mass, root mass and total biomass to transpiration loss. *RSWC*, soil water content. *FC*, field capacity. Data are shown as Mean ± SD, *n* = 4.

**FIGURE 6:**
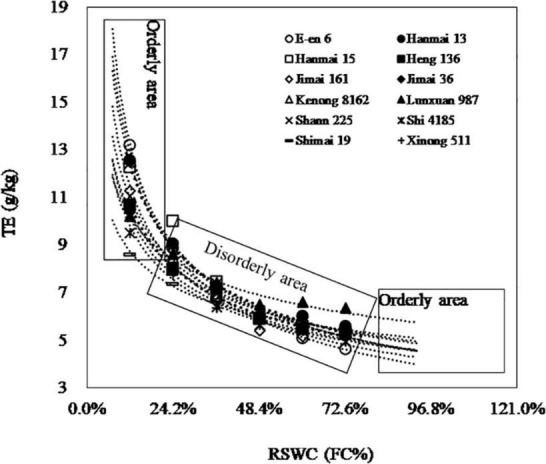
The rank of *TE* shoot among cultivars at different *RSWC* levels. The curves can be divided into orderly area where the rank of cultivars is stable when *RSWC* ≤ 12.1% or *RSWC* ≥ 72.6% and disorderly area where the rank is instable when 3% < *RSWC* < 18%, when comparing among number of cultivars. *TE _*shoot*_*, the ratio of shoot mass to transpiration loss. *RSWC*, soil water content. *FC*, field capacity. Data are shown as mean, *n* = 4.

Both cultivars and SWC showed a significant effect on *TE*, with the exception of their interaction (Table 1). The *TE _*shoot*_*, *TE*_root_ and *TE _*total*_* of the 12 cultivars de-creased with increasing *RSWC* in a power functional manner. The relationship was well described by Function 8 (Figures 5, 6). Here, we named this curvilinear relationship the *TE*–*RSWC* curve. According to the characteristics of the power function, *TE _*FC*_* could be understood as the *TE* of a certain cultivar measured at *SWC* at *FC*. The 12 cultivars showed very different responses of *TE* to the soil moisture gradient in magnitude. However, the response was similar in shape (Figures 5, 6). *TE _*FC*_* was between 7.5 and ∼11.4, 3.8 and ∼5.7, and 3.2 and ∼5.8, and b was between −0.5 and ∼−0.2, −0.6 and ∼−0.2, and −0.5 and ∼−0.2 for the *TE _*total*_*-*RSWC* curve, *TE _*shoot*_*-*RSWC* curve and *TE _*root*_*-*RSWC* curve, respectively.

The *TE*–*RSWC* curve was characterized by 4 points. Take *TE _*shoot*_*, which was most related to grain yield and most cared for by researchers, for example. First, *TE* varied depending on *RSWC*. Second, *TE* measured at any two adjacent *SWCs* had a positive correlation with each other (Table 2). Third, *TE*–*RSWC* curves could be divided into orderly areas, where *TE* became stable when *RSWC* went to extremely scarce (*RSWC* ≤ 12.1%) or plenty (*RSWC* ≥ 72.6%) levels, and disorderly areas, where *TE* was unstable when 12.1% < *RSWC* < 72.6% (Figure 6). Fourth, *TE* measured at 12.1% FC was negatively related to *TE _*FC*_*, and *TE* measured at 60.5 and 72.6% FC was positively related to *TE _*FC*_*. These 4 features of the *TE*–*RSWC* curve implied that comparison of *TE* among cultivars must and only be done under a given or similar *RSWC*.

**TABLE 2:** Correlation analysis between field measured yield (*GY*, grain yield; *BY*, biomass yield), water use efficiency (*WUE _*gy*_*, *WUE* at grain yield level; *WUE _*by*_*, *WUE* at biomass yield level), pot measured transpiration efficiency (*TE*) at different SWC levels (*TE* at x%, *TE* shoot measured at x% of *SWC*.) and *TE _*FC*_* (*TE* measured at field capacity, the parameter of the power function *TE* = *TE _*FC*_* × (*RSWC*)^b^.

	GY-0 mm	GY-150 mm	BY-0 mm	BY-150 mm	WUEgy-0 mm	WUEgy-150 mm	WUEby-0 mm	WUEby-150 mm	*TE* at 12.1%	*TE* at 24.2%	*TE* at 36.3%	*TE* at 48.4%	*TE* at 60.5%	*TE* at 72.6%	*TE _*FC*_*
GY-0mm	1														
GY-150mm	0.520	1													
BY-0mm	0.838**	0.749**	1												
BY-150mm	0.550	0.939**	0.693*	1											
WUEgy-0mm	0.920**	0.473	0.821**	0.523	1										
WUEgy-150mm	0.484	0.950**	0.720**	0.839**	0.462	1									
WUEby-0mm	0.705*	0.636*	0.888**	0.625*	0.865**	0.658*	1								
WUEby-150mm	0.521	0.903**	0.678*	0.897**	0.528	0.946**	0.681*	1							
*TE* at RSWC 12.1%	−0.708**	–0.545	−0.705*	–0.564	−0.742**	–0.537	−0.706*	–0.572	1						
*TE* at RSWC 24.2%	–0.329	–0.177	–0.269	–0.259	–0.533	–0.211	–0.508	–0.318	0.765**	1					
*TE* at RSWC 36.3%	–0.086	0.033	–0.026	0.031	–0.325	0.074	–0.272	0.103	0.358	0.623*	1				
*TE* at RSWC 48.4%	–0.010	–0.038	0.080	–0.031	–0.152	0.039	–0.058	0.091	0.054	0.159	0.657*	1			
*TE* at RSWC 60.5%	0.557	0.441	0.620*	0.466	0.458	0.494	0.504	0.574	–0.359	–0.124	0.394	0.677*	1		
*TE* at RSWC 72.6%	0.571	0.492	0.653*	0.534	0.443	0.465	0.465	0.527	–0.249	0.042	0.557	0.631*	0.868**	1	
*TE _*FC*_*	0.693*	0.601*	0.772**	0.646*	0.671*	0.610*	0.713**	0.691*	−0.778**	–0.537	0.067	0.471	0.834**	0.740**	1

Data are shown as correlation coefficient. * and ** means correlation is significant at the *P* = 0.05 and *P* = 0.01 levels, respectively. Data that is not followed by * means insignificant correlation between paired items.

The comparison of *TE* between cultivars could be categorized into two types. I) To compare between any two cultivars, the one with higher *TE _*FC*_* would show lower *TE* at lower *RSWC* and higher *TE* at higher *RSWC*. In other words, cultivars with lower *TE _*FC*_* would show higher *TE* at lower *RSWC* and lower *TE* at higher *RSWC*. In this situation, the intersection points between the *TE*–*RSWC* curve of any two cultivars would help to compare *TE* between cultivars (Figure 6). For example, to compare *TE* between Cultivar 1 and Cultivar 2, it is assumed that *TE*_1_ = *TE _*FC*1_* × *RSWC* ^*b*1^, *TE*_2_ = *TE _*FC*2_* × *RSWC* ^*b*2^. The *RSWC* at the intersection point was calculated as *RSWC*_*ip*_ = (*TE _*FC*2_*/*TE _*FC*1_*) ^ [1/(*b1* – *b2*)]. If a cultivar shows higher *TE* when *RSWC* is lower than the intersection point, it must show lower *TE* when *RSWC* is higher than the intersection point. II) To compare among large amounts of cultivars, the *RSWC* was recommended to be lower than 12.1% (near wilting point) or higher than 60.5% because the rank of cultivars became stable under these two conditions (Figure 6). The comparison results from the orderly area with a low *RSWC* would be opposite to the comparison results from the orderly area with a high *RSWC*.

**FIGURE 7:**
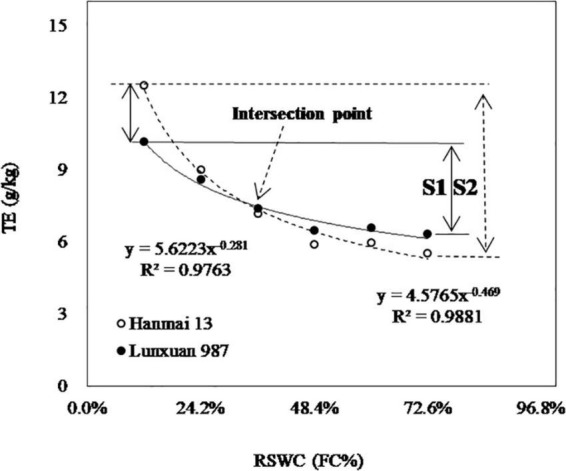
Sensitivity analysis and comparison of transpiration efficiency (*TE*) between any two cultivars, taking cultivars Hanmai 13 and Lunxuan 987 for example. The rank of sensitivity is inverse to the rank of *TE _*FC*_*. Sensitivity of Hanmai 13 (S2) is higher than that of Lunxuan 987 (S1). When comparing between two cultivars, *TE* of Hanmai 13 is higher than that of Lunxuan 987 to the left of the intersection point. The rank is reversed to the right of the intersection point. *RSWC*, soil water content. *FC*, field capacity. Data are means of 4 replicates at the same *RSWC*.

The *TE*–*RSWC* curve could be used to compare the sensitivity of *TE* to *RSWC* between cultivars. The existence of intersection point indicated that the sensitivity of *TE* to *RSWC* was different among cultivars. Simply, *TE _*FC*_* could be used directly to assess the sensitivity. Cultivars with higher *TE _*FC*_* might be less sensitive to *RSWC* because higher *TE _*FC*_* meant lower *TE* at lower *RSWC* and higher *TE* at higher *RSWC*. For example, the sensitivity of Cultivar Hanmai13, whose *TE _*FC*_* was less than that of Cultivar Lunxuan987, was more sensitive than that of the latter (Figure 7). From this viewpoint, the sensitivity of *TE* to *RSWC* was negatively related to *TE _*FC*_*. Accurately, the first-order derivative of the *TE*–*RSWC* curve function could be considered the sensitivity. However, the first-order derivative of a power function is still a power function. Thus, the sensitivity analysis became the same question as stated in the last paragraph, where the intersection point was introduced.

### Field yield and water use efficiency

GY and BY were both significantly affected by cultivar and irrigation. The interaction between cultivar and irrigation only showed remarkable effects on GY but not on BY (Table 3). Two irrigations (150 mm) resulted in 1853.14 kg/ha (*p* < 0.05) and 3035.10 kg/ha (*p* < 0.05) increases in GY and BY averaged over the 12 cultivars, respectively. Among all cultivars, Cultivar E-en6 showed minimal GY and BY under both irrigation conditions. This implied that E-en6 has no potential both in drought resistance and in high yield. That Shimai19 showed the highest GY under rain feeding indicated its ability to resist drought stress. Lunxuan 987, ranking 3rd under rainfed conditions and 1st under two irrigation treatments, either in GY or BY, was the most likely high-yielding cultivar under either drought or well-watered conditions.

**TABLE 3:** Effects of cultivar and irrigation on field observed grain yield (GY), Biomass yield (BY), evapotranspiration (ET), and water use efficiency at grain yield level (WUE _*gy*_) and biomass yield level (WUE _*by*_).

Source of variance		GY kg/ha	BY kg/ha	ET mm	WUEgy kg/ha/mm	WUEby kg/ha/mm
**Irrigation**	**Cultivar**					
0 mm	Er-en 6	4400.23 ± 413.34^d^	10290.11 ± 993.05^d^	320.2 ± 10.23^b^	13.78 ± 1.73^d^	32.22 ± 4.13^d^
	Hanmai 13	5857.03 ± 455.97^ab^	12611.27 ± 1103.53^abc^	320.03 ± 9.04^b^	18.29 ± 0.96^ab^	39.38 ± 2.67^abc^
	Hanmai 15	5694.86 ± 90.38^ab^	12354.96 ± 172.14^abc^	364.85 ± 16.79^a^	15.64 ± 0.97^bcd^	33.9 ± 1.19^cd^
	Heng 136	5207.13 ± 451.13^bc^	11537.19 ± 609.27^cd^	329.1 ± 38.89^b^	15.87 ± 0.92^bcd^	35.24 ± 2.47^bcd^
	Jimai 161	5343.94 ± 315.71^bc^	12916.08 ± 129.07^abc^	318.75 ± 9.41^b^	16.76 ± 0.59^abc^	40.54 ± 0.98^abc^
	Jimai 36	5436.79 ± 451.53^bc^	12766.38 ± 1388.4*abc*	315.18 ± 10.06^b^	17.25 ± 1.32^abc^	40.54 ± 4.82^abc^
	Kenong 8162	5894.57 ± 312.84^ab^	13048.95 ± 564.94^abc^	328.68 ± 4.75^b^	17.95 ± 1.21^ab^	39.72 ± 2.27^abc^
	Lunxuan 987	5932 ± 633.86^ab^	14087.14 ± 471.59^a^	330.43 ± 16.78^b^	18.03 ± 2.71^ab^	42.67 ± 1.6^a^
	Shann 225	4858.96 ± 708.21^cd^	12011.46 ± 1129.43^bc^	335.61 ± 7.49^b^	14.51 ± 2.36^cd^	35.8 ± 3.39^bcd^
	Shi 4185	5824.46 ± 81.63^ab^	13518.59 ± 1175.11^ab^	309.7 ± 19.77^b^	18.87 ± 1.42^a^	43.79 ± 5.08^a^
	Shimai 19	6359.96 ± 362.45^a^	13680.24 ± 1204ab	331.32 ± 13.77^b^	19.2 ± 0.89^a^	41.39 ± 4.79^ab^
	Xinong 511	6000.15 ± 318.91^ab^	12546 ± 1417.28^abc^	319.23 ± 10.65^b^	18.83 ± 1.61^a^	39.37 ± 5.03^abc^
150 mm	Er-en 6	5104.88 ± 472.55^e^	12772.09 ± 951.55^d^	429.23 ± 11.38^bcd^	11.9 ± 1.2^d^	29.77 ± 2.43^d^
	Hanmai 13	6284.84 ± 665.53^de^	14079.14 ± 1109.12^cd^	409.07 ± 13.27^e^	15.41 ± 2.09^c^	34.48 ± 3.49^bc^
	Hanmai 15	7671.87 ± 533.14^abc^	15759.04 ± 1408.69^abc^	437 ± 4.24^bc^	17.56 ± 1.21^abc^	36.06 ± 3.2^abc^
	Heng 136	7433.78 ± 899.31^abcd^	15761.48 ± 1094.26^abc^	414.77 ± 8.02^de^	17.9 ± 1.86^abc^	37.98 ± 1.97^ab^
	Jimai 161	7808.76 ± 965.47^ab^	16245.24 ± 1338.69^ab^	465.78 ± 9.58^a^	16.8 ± 2.39^bc^	34.91 ± 3.38^bc^
	Jimai 36	7995.33 ± 654.44^ab^	17067.1 ± 1276.26^ab^	420.03 ± 5.8^cde^	19.05 ± 1.81^ab^	40.66 ± 3.56^a^
	Kenong 8162	7007.99 ± 214.92^bcd^	15567.76 ± 1083.57^bc^	429.87 ± 4.99^bc^	16.3 ± 0.41^bc^	36.23 ± 2.72^abc^
	Lunxuan 987	8764.74 ± 294.12^a^	17659.1 ± 344.95^a^	435.42 ± 11.17^bc^	20.14 ± 1.06^a^	40.59 ± 1.85^a^
	Shann 225	6417.37 ± 202.07^cd^	13243.03 ± 209.58^d^	403.8 ± 6.20^e^	15.9 ± 0.67^bc^	32.8 ± 0.93^cd^
	Shi 4185	8116.28 ± 593.86^ab^	15851.06 ± 946.95^abc^	404.96 ± 2.06^e^	20.05 ± 1.54^a^	39.15 ± 2.54^ab^
	Shimai 19	7066.94 ± 1084.47^bcd^	15280.24 ± 717.85^bc^	429.66 ± 5.58^bcd^	16.45 ± 2.52^bc^	35.56 ± 1.46^abc^
	Xinong 511	7059.21 ± 1194.85^bcd^	15627.3 ± 1367.55^bc^	444.7 ± 20.95^b^	15.86 ± 2.45^bc^	35.17 ± 3.02^bc^
Cultivar	F value	7.75**	7.47**	4.75**	6.83**	5.78**
Irrigation	F value	142.01**	137.43 **	983.83**	0.127 NS	12.44**
Cultivar × Irrigation	F value	2.99**	1.56 NS	3.82**	2.54*	1.27 NS

* and ** means main effect is significant at the *P* = 0.05 and *P* = 0.01 levels, respectively. NS means non-significant at the *P* = 0.05 level. Data shown in Cultivar treatment is average over the 0 mm and 150 mm irrigation amount; Data shown in Irrigation treatment is average over the 12 cultivars. Different letters in the same column mean significant differences between treatments at 0.05 level.

Cultivars showed significant effects on both *WUE _*gy*_* and *WUE _*by*_*. Irrigation only showed a significant effect on *WUE _*by*_*. The combination only showed significant effects on *WUE _*gy*_* but not on *WUE _*by*_*. Two irrigation treatments decreased *WUE* by 2.02 kg/ha/mm (*p* < 0.05, averaged over 12 cultivars) compared to the rainfed treatment. The decrease might be attributed to the fact that 150 mm irrigation only resulted in a 99.90 mm increase in ET compared to rainfed treatment. This indicated that approximately 50.10 mm of irrigation was not used by wheat (Table 3). Among all cultivars, Cultivar E-en6 showed minimal *WUE _*gy*_* and *WUE _*by*_* under both irrigation conditions. Shimai19, Shi4185 and Xinong 511 showed the best *WUE* in the rainfed treatment, and Lunxuan987 and Shi4185 showed the best *WUE* in 150 mm irrigation.

### Relationship of seedling transpiration efficiency to whole seasonal growth and water use efficiency

**FIGURE 8:**
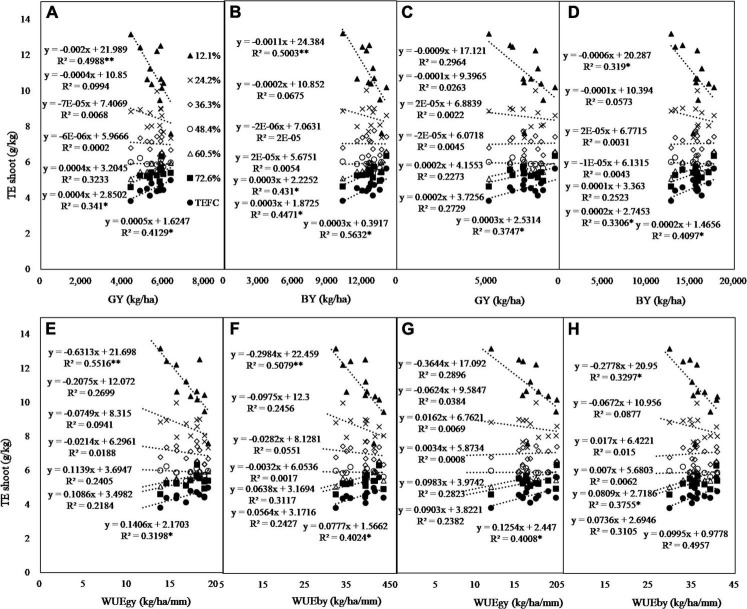
The regression of pot *TE _*shoot*_* to yield (*GY*, grain yield; *BY*, biomass yield) and water use efficiency (*WUE _*gy*_*, *WUE* at grain yield level; *WUE*_*by*_, *WUE* at biomass level) under rain-fed **(A,B,E,F)** and twice irrigation (150 mm, **C,D,G,H**). *TE _*FC*_*, *TE* measured at field capacity, *, ** means significant at the *P* = 0.05 and *P* = 0.01 levels, respectively.

The relationship of seedling *TE* to field-observed yield and *WUE* was dependent on the *SWC* level at which *TE* was observed (Figure 8 and Table 2). Linear correlation and regression analytical results indicated that *TE* measured at the lowest and highest *RSWC* was negatively and positively related to field yield and *WUE*, respectively, whether significant or not. The correlation decreased either with decreasing *RSWC* from 72.6% or with increasing *RSWC* from 12.1% to moderate *RSWC*. The minimal correlation coefficient appeared in *TE* measured at *RSWC* = 36.3% or *RSWC* = 48.4%. *TE* at *RSWC* = 12.1% was negatively correlated with *GY*, *BY*, *WUE _*gy*_* and *WUE _*by*_* under the rainfed treatment and not significantly correlated under 150 mm irrigation. There was almost no significant correlation of seedling *TE* to yield and *WUE* at other *RSWC* levels, except for the association of *TE* at *SWC* = 60.5% and at *RSWC* = 72.6% to *BY* under rainfed conditions. However, *TE _*FC*_*, which could be taken as *TE* measured at *SWC* of *FC*, was positively correlated with field yield and *WUE* either at grain yield or at biomass yield level, regardless of whether the wheat cultivar was irrigated.

## Discussion

### Response of transpiration efficiency to relative soil water content

The present study aimed to describe the response of *TE* to *RSWC* using a tightly sealed pot experiment. The results showed that *TE* decreased power functionally following the increase in *RSWC* gradients. The relationship can be described as *TE* = *TE _*FC*_* × (*RSWC*) *^b^*. In recent years, a large number of studies have focused on the variation in *TE* under various soil moisture conditions (Zhang et al., 1998; Jamalluddin et al., 2019). *TE* was usually found to decrease due to irrigation, precipitation and higher SWC (Zhang et al., 1998; Brueck et al., 2010; Sinclair, 2012). For instance, wheat *TE* at the dry matter level was found to be significantly higher under rainfed conditions than under irrigation in a 5-year experiment in northern Syria, an area with a typical Mediterranean climate (Zhang et al., 1998). Siahpoosh and Dehghanian (2012) reported the negative linear regression of *TE* against *ET*. The present results did not support their findings completely, especially in the shape of the regression line. Although many studies have focused on the response of *TE* to soil moisture, this is the first report on the power functional *TE*–*RSWC* curve to date within the bounds of our knowledge.

Soil water status is the most important factor that determines productivity and water consumption (Carter and Sheaffer, 1983; Flénet et al., 1996). Maintaining soil moisture at a higher level will help to harvest more yield. In arid and semiarid regions, various irrigation techniques (Zhou et al., 2021), water saving strategies (Wang et al., 2002), drought tolerant varieties (Kulathunga et al., 2021), bare soil mulching with straw or plastic film (Qin et al., 2022) and no-tillage (Guo et al., 2020; Peixoto et al., 2020) have been adopted and have been shown to be effective in improving crop yields. The common feature of all these yield-enhancing agronomic methods is the increase/maintenance in *SWC*. It must be mentioned that higher *SWC* also means higher evaporative loss in field crops and thus lower *TE* and *WUE*. This implies that the increased yield mainly results from the increased *Tr* (water uptake by plants, water use) but not the enhanced *TE*. This is not to say that breeding varieties with high *TE* are of no use. Varieties with high *TE* may have the potential to leave more water in the soil to maintain *SWC* at a higher level for later use (Christy et al., 2018). Thus, the best solutions for best wheat production exist in 3 choices, giving water as much as we can but not excessively, trying best to maintain the water in the soil available to the root system, and avoiding water loss through non-productive *E* as much as we can. This would help us to yield as much as possible in regions with either plenty of water or water shortages.

### Usage of the transpiration efficiency–relative soil water content curve

The *TE*–*RSWC* curve offers a promising method to screen cultivars/species for high yield and high *WUE* purposes. The second objective of the present study is to evaluate whether *TE* measured at the seedling stage can help to screen wheat cultivars with high yield and high *WUE* in the field. Although the *TE* of the present study was obtained from an experiment conducted at the seedling stage, the results indicated that *TE _*FC*_*, which was positively related to field yield and *WUE*, could be used as a surrogate for high yield and *WUE* (Figure 8). Additionally, the *TE* measured at *RSWC* ≤ 12.1% is also acceptable as an indicator of yield and *WUE* in rainfed conditions. However, *TE* measured at other *RSWC* values, i.e., 24.2, 36.3, 48.4, 60.5, and 72.6%, could not be used to represent field yield and *WUE*. This could be attributed to the instability of ranks among cultivars (Figure 6). A few other studies have confirmed that *TE* at the seedling stage can be used to represent seasonal *TE*. For example, Thapa et al. reported that the *TE* of sorghum was the same among six leaf, flag leaf, grain-filling and maturity stages (Thapa et al., 2017). The results of a four-water-level experiment in Iran showed that an increase in *TE* could improve the *WUE* in wheat genotypes (Siahpoosh and Dehghanian, 2012). The present study suggested that a cultivar with a higher *TE _*FC*_* may produce more *GY* and show higher *WUE* than a cultivar with a lower *TE _*FC*_*. This will greatly simplify the screening work for varieties with high *WUE*.

**FIGURE 9:**
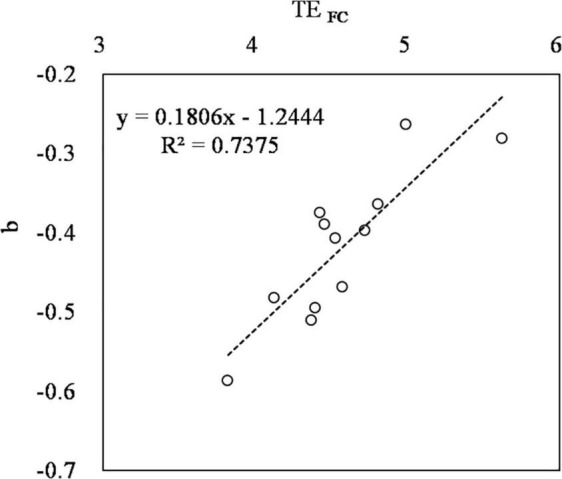
The relationship between parameter *TE _*FC*_* and b of the power function *TE* = *TE _*FC*_* × (*RSWC*)^b^ in *TE _*shoot*_* (transpiration efficiency of shoot, the ratio of shoot mass to transpiration loss).

The *TE*–*RSWC* curve offers a promising method to compare *TE* between cultivars or species not only applicable to wheat but also to other densely planted crops, such as millet and oats. However, conducting an experiment with many *SWC* levels would impede the application of this pot method. But the characteristics of the power function and the relationship between parameter b and *TE*_*FC*_ may help to simplify the experimental procedure. According to the feature of the power function *TE* = *TE _*FC*_* × (*RSWC*) ^b^, when *RSWC* = 1, *TE* = *TE _*FC*_*. That is, *TE _*FC*_* can be observed under soil moisture at field capacity or even saturated soil or water culture conditions. The situation is in accordance with Fletcher et al. (2018), who measured the *TE* of wheat cultivated with sufficient water. Additionally, parameter b is significantly related to *TE _*FC*_* (Figure 9), *b* = 0.1806 × *TE _*FC*_* - 1.2444 (*R*^2^ = 0.7375, *n* = 12, *p* ≤ 0.01). These two important features together make the work much easier than ever. Moreover, there is another advantage of high soil moisture conditions that should be addressed. Researchers can use open pots since the soil water content is no longer a limiting factor on the soil surface evaporation rate. *Tr* can be calculated as the *ET* of the planted pot minus the *ET* of the bare pot. This greatly simplifies the measuring process further.

There are also other possible uses of the *TE*–*RSWC* curve that should be addressed. The following usages were examined under the premise that the *TE*–*RSWC* curve was known. I) It could be used to estimate *TE* at any given *RSWC* (Function 7). This implied that we would be clear about the TE at any growing moment by real-time checking of the soil water status. II) It could be used to estimate Tr. Scientists from all over the world have tried methods to partition water used (ET) into transpiration (Tr) and soil evaporation (E) (Ritchie, 1972; Zhang et al., 1998). According to the present results and the definition of TE, Tr can be estimated as Tr = shoot mass/TE _*shoot*_, where shoot mass can be obtained by harvesting the aboveground part of the plant studied. TE shoot could be obtained with the *TE*–*RSWC* curve and *RSWC*. However, efforts are still needed to estimate long-term or seasonal Tr, such as the variability in the *TE*–*RSWC* curve among different growing stages.

### Uncertainties of the transpiration efficiency–relative soil water content curve

The present study provided an accurate measuring method of *TE* by restricting *E* to an extremely low level. The same pots without seedlings were tucked in with toothpicks made of bamboo with a diameter similar to that of wheat seedlings to test the proofness. The water loss rate was 0.03 ± 0.01 g⋅day^–1^⋅pot^–1^. The ratio of leakage to total *Tr* was approximately 10.81 ± 0.88%, 3.36 ± 0.34%, 1.83 ± 0.12%, 1.29 ± 0.06%, 1.09 ± 0.02%, and 1.01 ± 0.01% with increasing *RSWC* from 12.1 to 72.6%. Attention should be given to the fact that leakage will take a larger portion of the total Tr in the low moisture treatment. However, the error is easily eliminated because the leakage was the same independent of soil moisture. Researchers adopting this method are suggested to set out their study with a relative soil moisture no less than 24.2%.

Further studies should be conducted to certify the impacts of other factors that may affect the *TE*–*RSWC* curve. As is already known, variation in soil types (Ritchie, 1972; Tfwala et al., 2021), agronomic strategies such as fertilization (Hernandez et al., 2021) and organic amendments (Wang et al., 2021), growth regulators (Li et al., 2017, 2020), and environmental factors such as temperature (Vadez and Ratnakumar, 2016), atmospheric CO_2_ concentration (Tausz-Posch et al., 2012; Christy et al., 2018) and *VPD* [atmospheric vapor pressure deficit, (Vadez et al., 2014)] might change the *TE* of a given cultivar. Previous studies have also reported alterations in the ranking of *TE* among varieties resulting from some of these factors (Zhao et al., 2008; Fletcher et al., 2018). Setting out to study the effects of such factors on the *TE*–*RSWC* curve and the underlying mechanisms will help us to understand changes in *TE* due to exposure to various surroundings.

## Conclusion

Transpiration efficiency decreases power functionally following the increase in *RSWC*. The relationship can be described as *TE* = *TE _*FC*_* × (*SWC*)^b^. The parameter *TE _*FC*_* is the *TE* of a certain cultivar at *RSWC* = 1 (*SWC* = *FC*). The parameter b was intimately related to *TE _*FC*_* in a linear manner, i.e., *b* = 0.1806 × *TE _*FC*_* - 1.2444 (*R*^2^ = 0.7375, *n* = 12, *p* ≤ 0.01). The *TE*–*RSWC* curve can be used to compare *TE* between cultivars, to estimate transpiration loss of soil water, and to screen for high yield and high *WUE* varieties. Cultivars with higher TE _*FC*_ are expected to produce more *GY* with higher *WUE* under field conditions. The *TE*–*RSWC* curve can be briefly measured by planting crops under soil moisture at *FC* or with more than saturated soil moisture or even in water culture to obtain *TE _*FC*_* and then obtain parameter b according to the regression between *b* and *TE _*FC*_*. This curve may help greatly in screening for crop cultivars with high yield and high *WUE*.

## Data availability statement

The raw data supporting the conclusions of this article will be made available by the authors, without undue reservation.

## Author contributions

YQ and XZ designed the experiments and revised the manuscript. ML contributed to the formal analysis. DL and WQ contributed to the investigation and writing – original draft preparation. DL, YL, HY, and WL contributed to the resources. BD contributed to the data curation and project administration. YQ contributed to the writing – review and editing. XZ contributed to the supervision and funding acquisition. All authors read and approved the final manuscript.
